# Neutrophil Percentage‐to‐Albumin Ratio: Unveiling a New Perspective on Mortality Risk in Intensive Care Unit Asthma Patients—A Retrospective Cohort Study

**DOI:** 10.1155/mi/7147546

**Published:** 2026-03-18

**Authors:** Weide Lin, Junfan Chen, Bixia Lin

**Affiliations:** ^1^ Department of Anesthesiology, The First Hospital of Putian City, Putian, China; ^2^ Department of Medical Equipment Department, The First Hospital of Putian City, Putian, China; ^3^ Department of Ultrasonography, The First Hospital of Putian City, Putian, China

**Keywords:** asthma, ICU, nonlinear relationship, NPAR, retrospective cohort study

## Abstract

**Objectives:**

Asthma remains a prevalent diagnosis among intensive care unit (ICU) admissions, frequently linked to worsened patient outcomes. Therefore, identifying simple and effective indicators to predict the mortality risk of asthma patients in the ICU is particularly important. Given the unmet need for mortality biomarkers in critical asthma, this study specifically examined neutrophil percentage‐to‐albumin ratio’s (NPAR’s) correlation with ICU and in‐hospital death.

**Materials and Methods:**

We selected 1191 eligible asthma patients from the Medical Information Mart for Intensive Care‐IV (MIMIC‐IV) 3.0 database for analysis. This study applied a multivariate Cox regression model to explore the relationship between NPAR levels and the risk of mortality in the ICU and during hospitalization. Additionally, we used restricted cubic splines (RCSs) models to investigate the potential nonlinear dose–response relationship between NPAR levels and the risk of mortality in the ICU and during hospitalization.

**Results:**

Among the 1192 enrolled asthma cases, the cohort demonstrated a mean age of 58.1 ± 18.1 years, of which 38.7% were male. Asthma patients with higher NPAR faced substantially greater mortality risks during intensive care and hospitalization (hazard ratio [HR] range 1.75–2.14, *p*  < 0.01). The relationship between logarithmic transformation (ln) NPAR and ICU and in‐hospital mortality rates is nonlinear. Threshold effect analysis showed that when the ln NPAR level exceeded 0.5, the risk of patient mortality significantly increased (HR range 1.574–1.831, *p*  < 0.05). Additionally, subgroup analysis revealed no significant interactions.

**Conclusion:**

In ICU asthma patients, higher NPAR levels are associated with higher risks of ICU and in‐hospital mortality, emphasizing the importance of these findings for early identification and timely intervention in reducing the mortality risk of ICU asthma patients.

## 1. Introduction

Asthma, a prevalent chronic airway disorder, is characterized by airway obstruction, which is caused by a reduction in airway diameter. This narrowing of the airways is due to chronic inflammation, which leads to the accumulation and activation of immune cells in the airway walls [1]. In recent years, the number of patients admitted to the intensive care unit (ICU) due to asthma has been rising globally. Taking the United States as an example, approximately 25,000–50,000 asthma patients require ICU treatment each year [2]. The global prevalence, incidence, and mortality of asthma, as well as the economic burden it imposes, are all increasing significantly [3]. A study by Fahy et al. [4] found that in the sputum of patients during acute exacerbations of asthma, neutrophils are the predominant inflammatory cell type. They represent the major circulating leukocytes, mediating microbial clearance and tissue repair, and defend against foreign pathogens through phagocytosis or release of toxic substances from granules, constituting the body’s first‐line defense mechanism [5]. Additionally, asthma has been found to be associated with serum albumin levels [6], which may decrease due to various conditions such as inflammation, malnutrition, advanced liver disease, or malignant tumors [7]. Hypoalbuminemia, a marker of protein‐energy depletion, is associated with reduced respiratory (Resp) muscle strength and pulmonary function [8, 9]. In asthma patients, malnutrition prevalence is elevated and significantly correlates with poorer quality of life, increased exacerbation risk, prolonged hospitalization, and higher healthcare expenditures [10, 11]. A recent epidemiological study points out that the average mortality rate of asthma patients is 0.8% [12], therefore, proactive risk stratification and prompt intervention in high‐risk asthma cases are essential for mortality reduction.

The neutrophil percentage‐to‐albumin ratio (NPAR) is a cost‐effective and accessible biomarker that reflects both inflammatory status and nutritional conditions of the body [13, 14]. The mechanism of action lies in the fact that during inflammatory responses, neutrophils rapidly migrate to infected tissues, where they release reactive oxygen species and chemokines, contributing to vascular endothelial injury [15–17]. Meanwhile, serum albumin serves as a key biomarker of nutritional health [18], and its depletion frequently reflects underlying malnutrition or systemic inflammatory burden, both associated with adverse clinical outcomes [19]. Current studies have demonstrated that NPAR holds significant clinical value in prognostic evaluation for various diseases including liver cirrhosis, stroke, atrial fibrillation, and bladder cancer [20–23]. While NPAR’s prognostic value has been established in multiple pathologies, its clinical significance in asthma outcomes remains underexplored. Given the correlation between asthma and inflammation as well as nutritional status [6], our analysis based on the Medical Information Mart for Intensive Care‐IV (MIMIC‐IV) database explores the relationship between NPAR levels and asthma‐related mortality in patients.

## 2. Materials and Methods

### 2.1. Data Sources

This study employs the IRB–approved MIMIC‐IV database, a multidisciplinary initiative of MIT, Harvard Medical School, and Philips Healthcare. The project complies with the Declaration of Helsinki, and patient information in the MIMIC‐IV database has been anonymized. The use of data does not require approval from an ethics committee, therefore, no additional informed consent is needed from patients when using the MIMIC‐IV database. Our research methodology strictly follows the STROBE guidelines for observational epidemiology studies [24].

### 2.2. Study Design and Population

**Figure 1:**
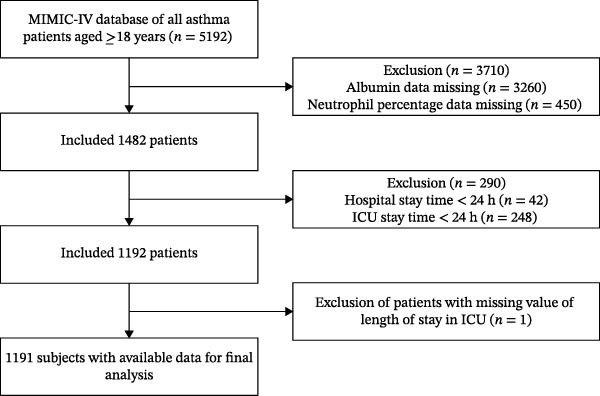
Schematic representation of the participant selection process and distribution of participant groups.

In this cohort study, we extracted data from participants over the age of 18 from the period of 2008–2022. We included 5192 asthma patients, excluded those with missing albumin and neutrophil percentage data, then excluded patients with ICU and hospital stays of less than 24 h, and excluded patients with missing ICU length of stay values, ultimately including 1191 patients. The study cohort exclusively comprised patients during their first ICU hospitalization. The enrollment process is shown in Figure 1.

### 2.3. Covariates and Outcome

Data extraction utilized PostgreSQL 13.9. The covariates considered in our analysis included: gender, age, race, insurance, heart rate, systolic blood pressure (SBP), diastolic blood pressure (DBP), Resp, pulse oximetry derived oxygen saturation (Spo_2_), glucose, hemoglobin (Hb), platelet count (PLT), lymphocyte count, neutrophil‐to‐lymphocyte ratio (NLR), acute kidney injury stage (AKI stage), β_2_‐agonist use during ICU stay (β_2_A‐use), myocardial infarction (MI), chronic heart failure (CHF), peripheral vascular disease (PVD), cerebrovascular disease (CVD), mechanical ventilation, Resp tract infection, urinary tract infection, and gastrointestinal infection. NPAR was computed as: (neutrophil % × 100)/albumin (g/dL) [25]. Primary endpoints included ICU and in‐hospital mortality.

### 2.4. Statistical Analysis

NPAR distribution was evaluated using Shapiro–Wilk test, prompting logarithmic transformation for normalization. Continuous variables were analyzed parametrically (mean ± SD; *t*‐test/ANOVA) or nonparametrically (median [IQR]; Kruskal–Wallis) based on distribution. Categorical variables used *χ* ^2^/Fisher’s exact tests with percentage reporting. Missing data (0.08%–1.43%) remained unprocessed given minimal impact.

Univariate and multivariate Cox regression models were constructed to evaluate NPAR’s association with ICU and in‐hospital mortality risk. We also constructed various statistical models to ensure the robustness of our study results. The multivariate models included clinically relevant variables as well as covariates with *p*‐values less than 0.1 in the univariate analysis. We employed sequentially adjusted multivariate models: an initial unadjusted model, Model 1 adjusted for age and gender, Model 2 additionally incorporating heart rate, glucose, CHF, lymphocyte count, and urinary tract infection, and Model 3 further adjusting for race, insurance, SBP, DBP, Resp, Spo_2_, Hb, PLT, NLR, AKI stage, β_2_A‐use, MI, PVD, CVD, mechanical ventilation, Resp tract infection, and gastrointestinal infection.

We evaluated NPAR’s mortality risk relationships through restricted cubic spline (RCS)–based nonlinear modeling complemented by threshold detection analyses.

We performed interaction tests and stratified analyses by age, sex, β_2_‐agonist use, and comorbidities (MI/CHF/CVD) to assess NPAR–mortality relationship consistency in asthma.

Statistical analyses were conducted in R 4.4.2 (R Foundation) and Free Statistics 2.0, applying two‐tailed *α* = 0.05 threshold for significance [26].

## 3. Results

### 3.1. Characteristics of the Participants

Out of 5192 participants over the age of 18, 4001 were excluded due to missing or unreliable data on albumin and neutrophil percentage, leaving 1191 participants for analysis. Table 1 presents participant demographics across NPAR tertiles, dividing the enrolled patients into a low group (NPAR < 1.87), a middle group (1.87 ≤ NPAR < 3.53), and a high group (NPAR ≥ 3.53). The average age of the 1191 participants was 58.1 ± 18.1, with 730 being female, and the majority being Caucasian and on medicare. Compared to the other two groups, patients with high NPAR levels had faster heart rates and Resp rates, lower blood pressure, higher blood sugar, and higher ICU and in‐hospital mortality rates.

**Table 1:** Baseline characteristics of participants.

Variables	Total (*n* = 1191)	T1 (*n* = 397) (<1.87)	T2 (*n* = 397) (≥1.87, <3.53)	T3 (*n* = 397) (≥3.53)	*p*‐Value
Gender, *n* (%)					0.235
Female	730 (61.3)	241 (60.7)	233 (58.7)	256 (64.5)	—
Male	461 (38.7)	156 (39.3)	164 (41.3)	141 (35.5)	—
Age (years)	58.1 ± 18.1	56.4 ± 18.4	59.7 ± 18.0	58.2 ± 17.8	0.038
Race/ethnicity, *n* (%)					0.575
White	668 (56.1)	219 (55.2)	224 (56.4)	225 (56.7)	—
Black	191 (16.0)	66 (16.6)	70 (17.6)	55 (13.9)	—
Other	332 (27.9)	112 (28.2)	103 (25.9)	117 (29.5)	—
Insurance, *n* (%)					0.378
Medicare	587 (50.0)	188 (47.8)	202 (52.1)	197 (50.1)	—
Private	295 (25.1)	99 (25.2)	103 (26.5)	93 (23.7)	—
Other	292 (24.9)	106 (27)	83 (21.4)	103 (26.2)	—
Heart rate (bpm)	109.9 ± 21.9	107.1 ± 22.6	109.3 ± 21.0	113.1 ± 21.9	<0.001
SBP (mm Hg)	148.8 ± 24.1	149.0 ± 24.7	151.8 ± 23.5	145.7 ± 23.7	0.002
DBP (mm Hg)	93.1 ± 20.7	95.2 ± 21.4	93.3 ± 20.3	90.8 ± 20.4	0.013
Resp (bpm)	29.4 ± 7.2	28.7 ± 7.5	29.3 ± 6.9	30.1 ± 7.1	0.027
Spo_2_ (%)	99.5 ± 1.2	99.5 ± 0.9	99.5 ± 1.0	99.4 ± 1.6	0.55
Glucose (mg/dL)	154 (119, 211)	138 (112, 182)	159 (124, 211)	166 (125, 229)	<0.001
Hb (g/dL)	11.8 ± 2.2	12.0 ± 2.3	11.8 ± 2.2	11.6 ± 2.2	0.022
PLT (×10^9^/L)	226.0 (161.0, 304.5)	196.0 (144.0, 265.0)	227.0 (162.0, 287.0)	260.0 (182.0, 351.0)	<0.001
Lymphocyte count (×10^9^/L)	1.2 (0.8, 1.9)	1.3 (0.8, 2.1)	1.2 (0.8, 1.7)	1.2 (0.7, 1.8)	0.015
NLR	7.7 (4.0, 15.2)	6.8 (3.4, 13.5)	8.2 (4.6, 16.4)	7.8 (4.2, 16.0)	0.008
AKI stage, *n* (%)					0.017
0	792 (66.5)	288 (72.5)	266 (67)	238 (59.9)	—
1	259 (21.7)	74 (18.6)	85 (21.4)	100 (25.2)	—
2	58 (4.9)	13 (3.3)	21 (5.3)	24 (6)	—
3	82 (6.9)	22 (5.5)	25 (6.3)	35 (8.8)	—
β_2_A‐use (%)					0.081
No	368 (31.0)	138 (34.8)	109 (27.5)	121 (30.5)	—
Yes	821 (69.0)	258 (65.2)	287 (72.5)	276 (69.5)	—
MI, *n* (%)					0.044
No	1040 (87.3)	360 (90.7)	342 (86.1)	338 (85.1)	—
Yes	151 (12.7)	37 (9.3)	55 (13.9)	59 (14.9)	—
CHF, *n* (%)					0.002
No	882 (74.1)	319 (80.4)	277 (69.8)	286 (72)	—
Yes	309 (25.9)	78 (19.6)	120 (30.2)	111 (28)	—
PVD, *n* (%)					0.281
No	1109 (93.1)	376 (94.7)	368 (92.7)	365 (91.9)	—
Yes	82 (6.9)	21 (5.3)	29 (7.3)	32 (8.1)	—
CVD, *n* (%)					0.005
No	1033 (86.7)	349 (87.9)	327 (82.4)	357 (89.9)	—
Yes	158 (13.3)	48 (12.1)	70 (17.6)	40 (10.1)	—
Mechanical ventilation, *n* (%)					<0.001
No	903 (75.8)	318 (80.1)	314 (79.1)	271 (68.3)	—
Yes	288 (24.2)	79 (19.9)	83 (20.9)	126 (31.7)	—
Respiratory tract infection, *n* (%)					<0.001
No	790 (66.3)	305 (76.8)	264 (66.5)	221 (55.7)	—
Yes	401 (33.7)	92 (23.2)	133 (33.5)	176 (44.3)	—
Urinary tract infection, *n* (%)					0.726
No	884 (74.2)	297 (74.8)	298 (75.1)	289 (72.8)	—
Yes	307 (25.8)	100 (25.2)	99 (24.9)	108 (27.2)	—
Gastrointestinal infection, *n* (%)					0.052
No	1140 (95.7)	376 (94.7)	388 (97.7)	376 (94.7)	—
Yes	51 (4.3)	21 (5.3)	9 (2.3)	21 (5.3)	—
ICU mortality, *n* (%)					<0.001
No	1063 (89.3)	374 (94.2)	364 (91.7)	325 (81.9)	—
Yes	128 (10.7)	23 (5.8)	33 (8.3)	72 (18.1)	—
In‐hospital mortality, *n* (%)					<0.001
No	1022 (85.8)	363 (91.4)	352 (88.7)	307 (77.3)	—
Yes	169 (14.2)	34 (8.6)	45 (11.3)	90 (22.7)	—

*Note:* Continuous variables are presented as mean ± SD or median (quartile), while categorical variables are presented as absolute numbers (percentages). PLT, platelet count; Spo_2_, pulse oximetry derived oxygen saturation; β_2_A‐use, β_2_‐agonist use during ICU stay.

Abbreviations: AKI stage, acute kidney injury stage; CHF, chronic heart failure; CVD, cerebrovascular disease; DBP, diastolic blood pressure; Hb, hemoglobin; ICU mortality, intensive care unit mortality; MI, myocardial infarction; NLR, neutrophil‐to‐lymphocyte ratio; PVD, peripheral vascular disease; Resp, respiratory; SBP, systolic blood pressure.

### 3.2. The Association Between NPAR and Mortality

Univariate analysis (Supporting Information 1: Table S1) and Kaplan–Meier survival curves (Supporting Information 2: Figure S1) consistently demonstrated significant associations between ln NPAR and both ICU and in‐hospital mortality among mechanically ventilated ICU patients.

The results of the multivariate Cox regression analysis are shown in Table 2. A correlation between ln NPAR and the risk of ICU and in‐hospital mortality was found in both unadjusted and all adjusted models (hazard ratio [HR] range 1.26–1.44, *p*  < 0.05). Patients with NPAR ≥ 3.53 demonstrated significantly increased ICU and in‐hospital mortality across all adjusted models (HR range 1.75–2.14, *p*  < 0.01). The RCS model revealed a nonlinear relationship between ln NPAR and the risks of both ICU and in‐hospital mortality (*p*  < 0.05; Figure 2). Threshold analysis identified 0.5 as the inflection point, beyond which each unit increase in ln NPAR elevated ICU mortality risk by 57.4% and in‐hospital mortality by 83.1% (Table 3).

Figure 2(A) Nonlinear dose–response relationship between ln NPAR and ICU mortality. (B) Nonlinear dose–response relationship between ln NPAR and in‐hospital mortality. Both adjustment factors included gender, age, race, insurance, heart rate, SBP, DBP, Resp, Spo_2_, Hb, glucose, AKI stage, MI, CHF, PVD, and CVD. The blue line and green area represent the estimated values and their corresponding 95% confidence intervals, respectively.

**Figure:**
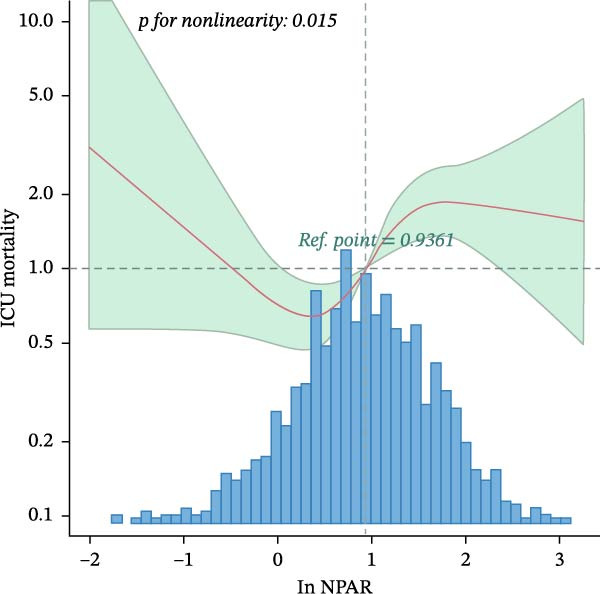
(A)

**Figure:**
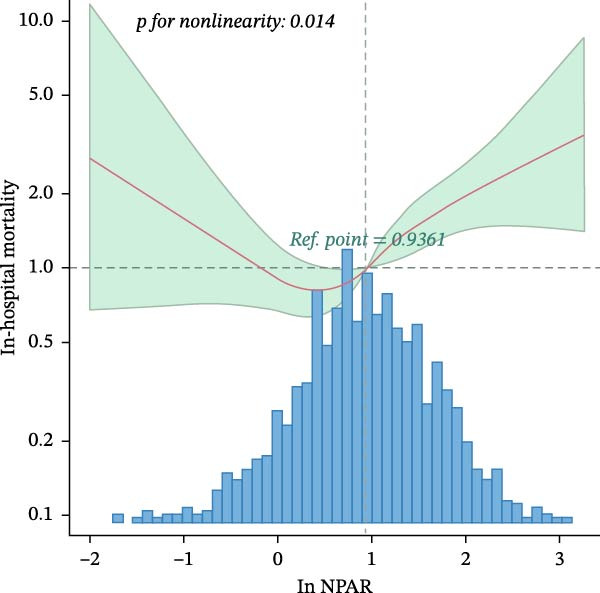
(B)

**Table 2:** Relationships between NPAR, ICU mortality, and in‐hospital mortality in different models.

Variable	Crude model		Model Ⅰ		Model Ⅱ		Model Ⅲ	
	HR (95% CI)	*p*‐Value	HR (95% CI)	*p*‐Value	HR (95% CI)	*p*‐Value	HR (95% CI)	*p*‐Value
ICU mortality								
ln NPAR	1.38 (1.1–1.74)	0.005	1.44 (1.13–1.82)	0.003	1.3 (1.02–1.65)	0.031	1.31 (1.01–1.7)	0.044
Tertiles								
NPAR < 1.87	Ref	—	Ref	—	Ref	—	Ref	—
1.87 ≤ NPAR < 3.53	1.03 (0.6–1.75)	0.921	1.02 (0.6–1.73)	0.953	1.01 (0.58–1.74)	0.977	1.01 (0.57–1.77)	0.974
NPAR ≥ 3.53	2.12 (1.33–3.4)	0.002	2.14 (1.33–3.44)	0.002	1.89 (1.15–3.1)	0.012	2.08 (1.23–3.52)	0.007
*p* for trend	—	<0.001	—	<0.001	—	0.003	—	0.002
In‐hospital mortality								
ln NPAR	1.39 (1.14–1.69)	0.001	1.41 (1.15–1.72)	0.001	1.34 (1.1–1.64)	0.003	1.26 (1.02–1.57)	0.034
Tertiles								
NPAR < 1.87	Ref	—	Ref	—	Ref	—	Ref	—
1.87 ≤ NPAR < 3.53	1.16 (0.75–1.82)	0.504	1.11 (0.71–1.74)	0.645	1.12 (0.71–1.77)	0.621	0.99 (0.62–1.59)	0.97
NPAR ≥ 3.53	2.04 (1.37–3.03)	<0.001	2 (1.34–2.97)	0.001	1.93 (1.29–2.89)	0.001	1.75 (1.13–2.7)	0.012
*p* for trend	—	<0.001	—	<0.001	—	<0.001	—	0.005

*Note:* Crude model was not adjusted. Model 1 was adjusted for age + gender. Model 2 was adjusted for Model 1 + heart rate + glucose + CHF + lymphocyte count + urinary tract infection. Model 3 was adjusted for Model 2 + race + insurance + SBP + DBP + Resp + Spo_2_ + Hb + PLT + NLR + AKI stage + β_2_A‐use + MI + PVD + CVD + mechanical ventilation + respiratory tract infection + gastrointestinal infection. PLT, platelet count; Spo_2_, pulse oximetry derived oxygen saturation.

Abbreviations: AKI stage, acute kidney injury stage; CHF, chronic heart failure; CVD, cerebrovascular disease; DBP, diastolic blood pressure; Hb, hemoglobin; MI, myocardial infarction; NLR, neutrophil‐to‐lymphocyte ratio; PVD, peripheral vascular disease; Resp, respiratory; SBP, systolic blood pressure.

**Table 3:** Threshold effect analysis of ln‐transformed NPAR levels on ICU mortality and in‐hospital mortality.

ICU mortality				In‐hospital mortality			
Turning point	HR	95% CI	*p*‐Value	Turning point	HR	95% CI	*p*‐Value
ln NPAR <0.5	0.212	0.044, 1.008	0.0512	ln NPAR <0.5	0.541	0.209, 1.403	0.2064
ln NPAR ≥0.5	1.574	1.082, 2.289	0.0176	ln NPAR ≥0.5	1.831	1.332, 2.518	<0.001
Likelihood ratio test	—	—	0.03	Likelihood ratio test	—	—	0.009

*Note:* Both adjustment factors included gender, age, race, insurance, heart rate, SBP, DBP, Resp, Spo_2_, Hb, glucose, AKI stage, MI, CHF, PVD, and CVD.

Abbreviations: CI, confidence interval; HR, hazard ratio.

### 3.3. Subgroup Analysis

Subgroup analyses by age, sex, β_2_A‐use, MI, CHF, and CVD revealed consistent NPAR–mortality associations (all interaction *p*  > 0.05, Supporting Information 3: Figure S2).

## 4. Discussion

This US retrospective cohort study of adult asthma patients demonstrated that elevated NPAR levels significantly predicted mortality risk, with threshold effects observed above a defined cutoff. Subgroup analyses confirmed consistent associations across all strata.

The occurrence of asthma is closely related to the complex interactions between immune cells, which are often triggered when individuals are exposed to specific environmental factors early in life [1]. Lung autopsy findings from fatal asthma cases provided foundational histopathological data [27, 28]. These studies revealed that airway inflammation is a key feature of severe and fatal asthma [29]. However, with the development and application of bronchoscopy techniques such as airway lavage and biopsy, scientists have found that airway inflammation is present in all stages of asthma, indicating that airway inflammation is a common clinical feature of the disease [30, 31]. Furthermore, further research on the histological characteristics of asthmatic airways has provided new insights into the causes of airway inflammation. Globally, asthma is responsible for approximately 180,000 deaths per year [3]. Currently, biomarkers involved in asthma management include IgE, eosinophils, and cytokines, among others [32]. These biomarkers aid in the diagnosis of asthma, assessment of disease activity, and monitoring of treatment effects; however, they have certain limitations in predicting patient mortality risks. Therefore, the development of new biomarkers holds significant clinical importance for understanding the prognosis of asthma patients in the ICU.

This novel inflammatory–nutritional biomarker (NPAR) synergistically reflects neutrophil‐driven inflammation and albumin‐modulated immunity [33], with elevated levels predicting mortality potentially through amplified inflammatory cascades. In our previous study, we established a significant association between elevated NPAR levels and increased mortality risks over both short (30‐day) and long (365‐day) periods in patients with iron‐deficiency anemia (IDA), revealing an inverse “L” shaped relationship between ln NPAR levels and mortality risks [34]. The commonalities between IDA and asthma are that both are related to inflammation and nutrition to some extent. Building on these findings, the current study shifts focus to asthma patients, a distinct patient population from the IDA cohort in our prior research. We observed a nonlinear relationship between ln NPAR and ICU as well as in‐hospital mortality rates, which differs from the inverse “L” shaped pattern found in the IDA study. The lack of significant interactions in subgroup analyses across both studies underscores the robustness of NPAR as a prognostic marker, irrespective of the underlying disease. However, the differing patterns and thresholds of NPAR’s association with mortality risk between IDA and asthma patients highlight the disease‐specific nuances in the impact of NPAR. This distinction not only enriches our understanding of NPAR’s role in different clinical contexts but also has implications for targeted interventions and patient management strategies in these two distinct patient populations. Thus, the current study contributes new insights by extending the investigation of NPAR’s prognostic value to asthma patients and elucidating the unique relationship between NPAR levels and mortality risks in this group, thereby avoiding redundancy with our previous work while advancing the scientific discourse on NPAR’s clinical significance.

A study on COPD patients in the United States indicated that NPAR is related to adverse prognosis in COPD patients, possibly due to inflammation induction [35]. A prospective multicenter study in China found that NPAR showed high accuracy and reliability in predicting spontaneous bacterial peritonitis [36]. Additionally, studies have shown that NPAR elevation, previously linked to sepsis mortality [37, 38], serves as a dynamic indicator of immune‐inflammatory equilibrium and pathological progression. Numerous studies have shown that NPAR, as an important clinical indicator, is closely related to the mortality risk of patients with various diseases, such as cardiovascular diseases [39–42], infectious diseases [43], CVDs [44, 45], or oncology [46], where NPAR also shows potential. The elevated NPAR observed in this study potentially mirrors intensified inflammatory cascades in asthma, contributing to airway structural changes and functional decline that ultimately elevate mortality risk. In summary, our study shows that NPAR is closely related to the prognosis of asthma patients in the ICU. Medical staff can assess the risk level based on the NPAR levels of patients at admission. Asthma patients with higher NPAR levels should be given special attention by doctors.

Although this study provides valuable insights, it is not without limitations. First, the retrospective design may not fully control for potential confounding factors and cannot directly establish a causal relationship between NPAR and prognosis. Second, data on the dosing and duration of β_2_‐agonists and theophylline were unavailable. However, stratified analyses based on the timing of medication use confirmed the robustness of the association between NPAR and mortality risk. This study is the first to reveal the prognostic value of NPAR in a real‐world severe asthma cohort, providing a key basis for future prospective studies. Future research should further explore the clinical significance of NPAR through multicenter designs combined with mechanistic studies.

## 5. Conclusion

Our study indicates that higher NPAR levels are positively correlated with increased risk of ICU and in‐hospital mortality. There is a nonlinear relationship between ln NPAR levels and the risk of ICU and in‐hospital mortality among ICU asthma patients, with higher NPAR levels associated with increased risk of mortality, particularly when ln NPAR exceeds 0.5, the risk increases significantly.

## Author Contributions

**Weide Lin:** conceptualization, formal analysis, survey, methodology, software, visualization, writing – original draft. **Junfan Chen:** data curation, survey, software, validation. **Bixia Lin:** conceptualization, funding acquisition, project administration, resources, supervision, validation, writing – review and editing.

## Funding

This study was not funded by any institution.

## Ethics Statement

The establishment of the MIMIC‐IV database was approved by the Institutional Review Boards of BIDMC and the MIT. This project adheres to the Declaration of Helsinki, and patient information has been anonymized, thus, the use of data from this database does not require ethics committee approval, nor does it necessitate additional informed consent from patients. This study strictly complies with all applicable laws and regulations to ensure its legality and ethicality.

## Consent

The authors have nothing to report.

## Conflicts of Interest

The authors declare no conflicts of interest.

## Supporting Information

Additional supporting information can be found online in the Supporting Information section.

## Supporting information


**Supporting Information 1** Table S1. Univariate analysis of ICU and in‐hospital mortality rates among patients with asthma. This table presents the results of univariate analysis on risk factors associated with ICU mortality and in‐hospital mortality in asthma patients, detailing the various risk factors and their correlations with mortality rates.

**Supporting Information 2** Figure S1. (A) Kaplan–Meier survival analysis of NPAR and ICU mortality in patients with asthma. (B) Kaplan–Meier survival analysis of NPAR and in‐hospital mortality in patients with asthma. Patients were stratified into tertiles based on NPAR thresholds: T1: NPAR < 1.87; T2: 1.87 ≤ NPAR < 3.53; T3: NPAR ≥ 3.53. Log‐rank test indicated significant survival differences between groups (*p* < 0.001), with T3 associated with the poorest prognosis. Baseline characteristics were balanced, with 397 patients at risk per group.

**Supporting Information 3** Figure S2. Subgroup analysis of the association between NPAR and ICU mortality and in‐hospital mortality in asthma patients. Figure S2 conducts a subgroup analysis of the association between the neutrophil percentage‐to‐albumin ratio (NPAR) and ICU mortality and in‐hospital mortality in asthma patients, further exploring the influence of NPAR in different patient populations with asthma.

## Data Availability

The data that support the findings of this study are available in MIMIC‐IV at https://mimic.physionet.org/ (Reference Number 10.13026/hxp0‐hg59).
