# Supplementary material for: Neutrophil Percentage‐to‐Albumin Ratio: Unveiling a New Perspective on Mortality Risk in Intensive Care Unit Asthma Patients—A Retrospective Cohort Study
Source: Mediators Inflamm. 2026 Mar 18;2026:7147546. doi: 10.1155/mi/7147546 (PMC13140161; doi:10.1155/mi/7147546)
Supplement: Supplementary file 1 — Supporting Information 1 Table S1. Univariate analysis of ICU and in‐hospital mortality rates among patients with asthma. This table presents the results of univariate analysis on risk factors associated with ICU mortality and in‐hospital mortality in asthma patients, detailing the various risk factors and their correlations with mortality rates. [file MI-2026-7147546-s002.docx]

**TABLE S1** | Univariate analysis of in-hospital and ICU mortality rates among patients with asthma.

| **Variables** | **ICU mortality** | | **In-hospital mortality** | |
| --- | --- | --- | --- | --- |
|  | **HR(95%CI)** | ***P-*value** | **HR(95%CI)** | ***P-*value** |
| **Gender** | 0.82 (0.56,1.18) | 0.277 | 0.77 (0.56,1.07) | 0.119 |
| **Age** | 1.02 (1.01,1.03) | 0.001 | 1.02 (1.01,1.03) | < 0.001 |
| **Race/Ethnicity** |  |  |  |  |
| White | Ref |  | Ref |  |
| Black | 0.95 (0.57,1.58) | 0.841 | 0.92 (0.58,1.45) | 0.709 |
| Other | 0.95 (0.65,1.41) | 0.816 | 1.09 (0.78,1.53) | 0.605 |
| **Insurance** |  |  |  |  |
| Medicare | Ref |  | Ref |  |
| Private | 0.8 (0.51,1.24) | 0.31 | 0.66 (0.44,0.98) | 0.041 |
| Other | 0.76 (0.49,1.19) | 0.236 | 0.68 (0.46,0.99) | 0.047 |
| **Heart rate** | 1.008 (1.0009,1.0151) | 0.026 | 1.0074 (1.001,1.0139) | 0.024 |
| **SBP** | 0.9942 (0.9872,1.0013) | 0.109 | 0.9958 (0.9894,1.0022) | 0.197 |
| **DBP** | 1.0012 (0.9929,1.0096) | 0.781 | 0.9969 (0.9894,1.0044) | 0.418 |
| **Resp** | 0.9918 (0.9694,1.0147) | 0.477 | 1.0006 (0.9811,1.0204) | 0.953 |
| **Spo_2_** | 0.96 (0.84,1.1) | 0.528 | 0.95 (0.82,1.09) | 0.463 |
| **Hb** | 0.95 (0.88,1.03) | 0.2 | 0.97 (0.91,1.04) | 0.462 |
| **PLT** | 1.0007 (0.9994,1.002) | 0.27 | 1.0004 (0.9993,1.0016) | 0.465 |
| **Lymphocyte count** | 1.02 (1,1.04) | 0.055 | 1.02 (1.01,1.04) | 0.006 |
| **NLR** | 1.0045 (0.9988,1.0102) | 0.121 | 1.0043 (0.9987,1.0099) | 0.129 |
| **Glucose** | 1.0019 (1.0007,1.0031) | 0.002 | 1.0009 (1,1.0019) | 0.042 |
| **AKI stage** |  |  |  |  |
| 0 | Ref |  | Ref |  |
| 1 | 2.3 (1.51,3.5) | < 0.001 | 2.09 (1.46,2.99) | < 0.001 |
| 2 | 1.69 (0.92,3.09) | 0.089 | 2.08 (1.21,3.59) | 0.008 |
| 3 | 2.08 (1.23,3.5) | 0.006 | 2.63 (1.69,4.09) | < 0.001 |
| **β₂A-use** | 0.62 (0.42,0.93) | 0.021 | 0.93 (0.67,1.29) | 0.648 |
| **MI** | 1.38 (0.9,2.11) | 0.141 | 1.83 (1.27,2.65) | 0.001 |
| **CHF** | 1.38 (0.95,2) | 0.087 | 1.35 (0.98,1.85) | 0.064 |
| **PVD** | 1.04 (0.59,1.83) | 0.901 | 1.33 (0.82,2.18) | 0.248 |
| **CVD** | 0.9 (0.56,1.46) | 0.681 | 1.09 (0.71,1.67) | 0.692 |
| **Mechanical ventilation** | 1.05 (0.73,1.5) | 0.807 | 1.48 (1.08,2.03) | 0.015 |
| **Respiratory tract infection** | 0.81 (0.57,1.16) | 0.249 | 1.37 (1.01,1.86) | 0.044 |
| **Urinary tract infection** | 0.55 (0.35,0.86) | 0.009 | 0.55 (0.38,0.81) | 0.002 |
| **Gastrointestinal infection** | 0.31 (0.04,2.2) | 0.24 | 0.6 (0.22,1.61) | 0.309 |
| **ln NPAR** | 1.38 (1.1,1.74) | 0.005 | 1.39 (1.14,1.69) | 0.001 |

**Abbreviations:** SBP, systolic blood pressure; DBP, diastolic blood pressure; Resp, respiratory; Spo_2,_ pulse oximetry derived oxygen saturation; Hb, hemoglobin; PLT, Platelet count; NLR, Neutrophil-to-Lymphocyte Ratio; AKI stage, acute kidney injury stage; **β₂A-use,** β₂-agonist use during ICU stay; MI, myocardial infarction; CHF, chronic heart failure; PVD, peripheral vascular disease; CVD, cerebrovascular disease; ln NPAR, natural logarithm neutrophil percentage-to-albumin ratio.
