# Supplementary figures and images for: Neutrophil Percentage‐to‐Albumin Ratio: Unveiling a New Perspective on Mortality Risk in Intensive Care Unit Asthma Patients—A Retrospective Cohort Study
Source: Mediators Inflamm. 2026 Mar 18;2026:7147546. doi: 10.1155/mi/7147546 (PMC13140161; doi:10.1155/mi/7147546)

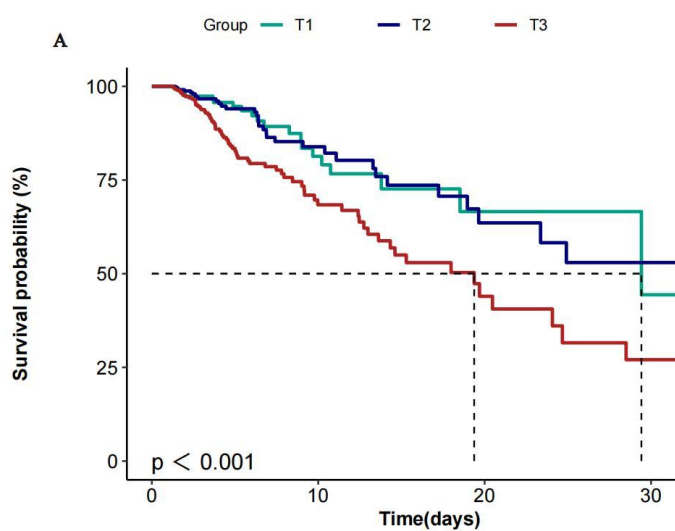

| Tertiles | Number at risk |    |    |   |
|----------|----------------|----|----|---|
| T1       | 397            | 36 | 10 | 2 |
| T2       | 397            | 52 | 14 | 6 |
| T3       | 397            | 53 | 13 | 6 |

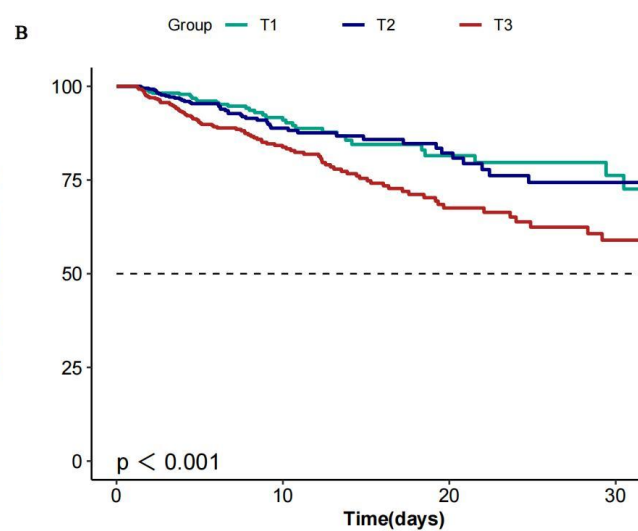

| Tertiles | Number at risk |     |    |    |
|----------|----------------|-----|----|----|
| T1       | 397            | 129 | 48 | 21 |
| T2       | 397            | 150 | 61 | 23 |
| T3       | 397            | 186 | 67 | 33 |

Supplement: Supplementary file 2 — Supporting Information 2 Figure S1. (A) Kaplan–Meier survival analysis of NPAR and ICU mortality in patients with asthma. (B) Kaplan–Meier survival analysis of NPAR and in‐hospital mortality in patients with asthma. Patients were stratified into tertiles based on NPAR thresholds: T1: NPAR < 1.87; T2: 1.87 ≤ NPAR < 3.53; T3: NPAR ≥ 3.53. Log‐rank test indicated significant survival differences between groups (p < 0.001), with T3 associated with the poorest prognosis. Baseline characteristics were balanced, with 397 patients at risk per group. [file MI-2026-7147546-s003.pdf]

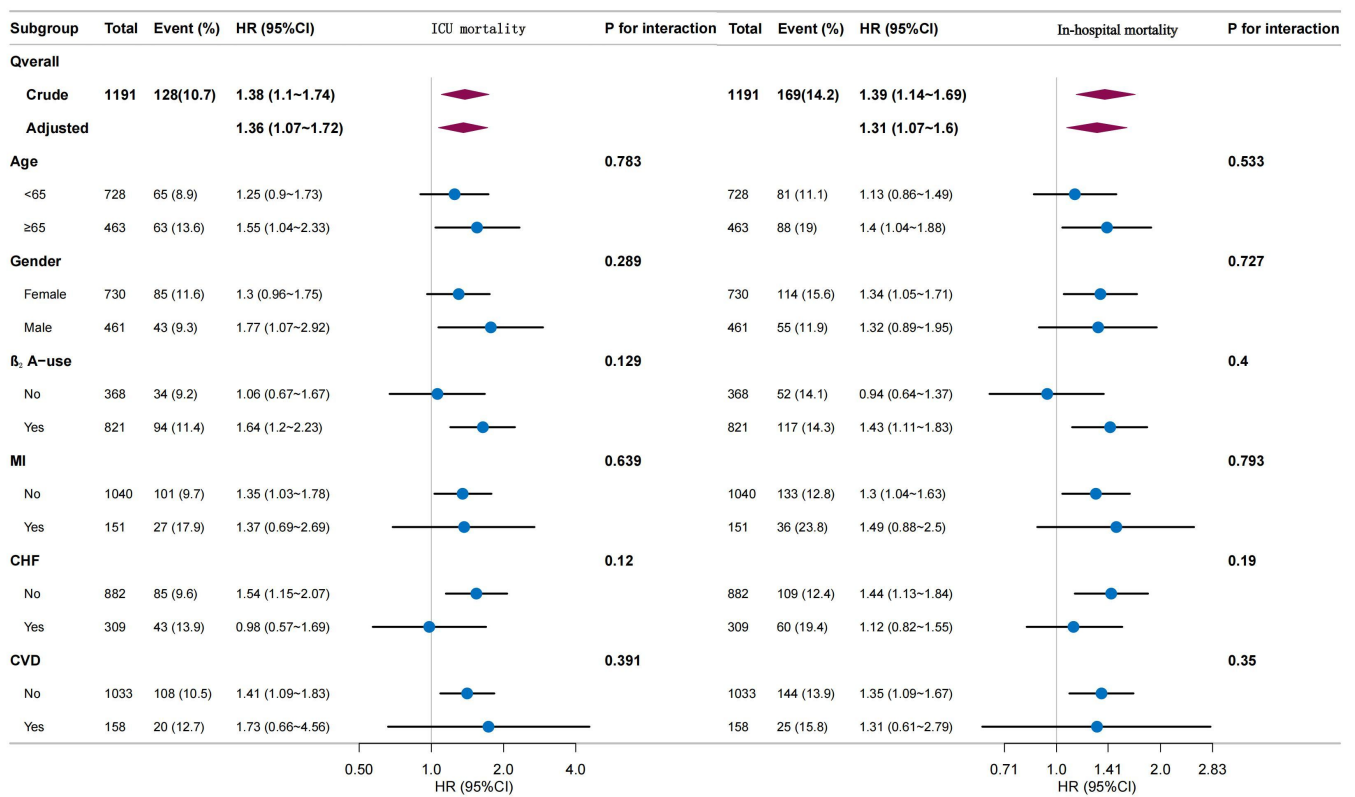

Supplement: Supplementary file 3 — Supporting Information 3 Figure S2. Subgroup analysis of the association between NPAR and ICU mortality and in‐hospital mortality in asthma patients. Figure S2 conducts a subgroup analysis of the association between the neutrophil percentage‐to‐albumin ratio (NPAR) and ICU mortality and in‐hospital mortality in asthma patients, further exploring the influence of NPAR in different patient populations with asthma. [file MI-2026-7147546-s001.pdf]
